# Intrauterine inflammation induced white matter injury protection by fibrinogen-like protein 2 deficiency in perinatal mice

**DOI:** 10.1038/s41390-020-01211-w

**Published:** 2020-10-19

**Authors:** Di Zhan, Cai Zhang, Wenjun Long, Lan Wei, Shengjuan Jin, Caiqi Du, Zhuxi Li, Shusen Guo, Lianjing Huang, Qin Ning, Xiaoping Luo

**Affiliations:** 1 Department of Pediatrics, Tongji Hospital, Tongji Medical College, Huazhong University of Science and Technology, Wuhan, Hubei China; 2 Department of Infectious Diseases, Tongji Hospital, Tongji Medical College, Huazhong University of Science and Technology, Wuhan, Hubei China

## Abstract

**Background:**

White matter injury (WMI) induced by intrauterine inflammation can cause adverse neurological outcomes. Fibrinogen-like protein 2 (FGL2)/fibroleukin is an important trigger of inflammatory responses and is involved in some cerebral diseases. However, the role of FGL2 in intrauterine inflammation-induced WMI remains unclear.

**Methods:**

Lipopolysaccharide (LPS) was intraperitoneally injected into wild-type and FGL2 knockout mice to induce intrauterine inflammation. Body weight and brain weight of offspring were monitored. Major basic protein (MBP) expression was evaluated to demonstrate the myelination of offspring. To investigate the regulatory mechanism of FGL2, cytokine expression, microglial polarization, and the activation of mitogen-activated protein kinase (MAPK) signaling pathway in the offspring were analyzed.

**Results:**

Upon LPS exposure, FGL2 knockout offspring showed a significant increase in body weight loss. MBP reduction induced by LPS was prevented in FGL2 knockout offspring. Expression levels of proinflammatory cytokines interleukin-1β (IL-1β) and tumor necrosis factor-α, and M1 marker CD86 were suppressed, while the expression levels of anti-inflammatory cytokines IL-10 and M2 marker CD206 were increased. FGL2 deficiency significantly inhibited the phosphorylation of p38MAPK and c-Jun N-terminal kinase (JNK) protein.

**Conclusions:**

FGL2 deficiency can ameliorate WMI induced by intrauterine inflammation, reducing inflammatory cascade and improving hypomyelination, through the regulation of microglial polarization and MAPK signaling pathways.

**Impact:**

- Intrauterine inflammation induces WMI leading to severe neurological sequelae. FGL2 plays an important role in the progression of WMI induced by intrauterine inflammation.
- FGL2 deficiency can protect against WMI by inhibiting p38 MAPK and JNK phosphorylation, regulating microglia polarization, and reducing inflammation response.
- FGL2 could be a novel molecular target for protecting against WMI induced by intrauterine inflammation.

## Introduction

White matter injury (WMI) is a common form of immature brain injury, and one of the leading causes of long-term neurological deficits, such as cognitive deficit, mental retardation, and even cerebral palsy (CP). Periventricular leukomalacia is a type of WMI characterized by diffuse demyelination of periventricular white matter. Clinical studies indicate that intrauterine inflammation, such as chorioamnionitis, is a risk factor for both CP and cystic periventricular leukomalacia,^1^ and increases the risk of CP in term and preterm infants.^2–4^ However, the pathogenesis of intrauterine inflammation-induced WMI during brain development is complex and not fully understood.

Intrauterine inflammation can cause inflammatory response, ischemia–hypoxia, and oxidative stress, and so on, leading to WMI.^5^ Cytokine is one of the strong link factors in the inflammatory response. Intrauterine inflammation causes the production and release of proinflammatory cytokines, thereby increasing the permeability of the blood–brain barrier (BBB). Cytokines enter the brain through the damaged BBB, which not only directly damage the immature brain but also induce cerebral inflammatory cascade and aggravate secondary brain injury.^6^ Microglia, the brain-resident macrophages, are involved in immune surveillance, oligodendrogenesis, and neurogenesis during brain development.^7^ In the inflammatory state, microglia are the main producers of cytokines. Proinflammatory factors can polarize microglia to the M1 type, and further promote the secretion of proinflammatory chemokines, cytokines, and coagulation factors.^8^ In turn, activated coagulation factors enhance the inflammatory response.^9^ Eventually, immature oligodendrocytes and neurons are damaged by proinflammatory factors, resulting in white matter damage and neurological disorders. Alternatively, the M2-polarized microglia are associated with the inhibition of inflammation and the recovery from damage. The M1/M2 polarization of microglia plays a crucial role in the balance between exacerbation and remission of neuroinflammation. But this dynamic balance is regulated by multiple factors and the mechanism is not completely clear.

Fibrinogen-like protein 2 (FGL2), a member of the fibrinogen super family, has been shown to be strongly correlated with cerebral diseases, such as acute cerebral ischemic–reperfusion injury, intracerebral hemorrhage, and traumatic brain injury.^10–12^ However, the detailed mechanisms are still unknown. FGL2 is usually expressed in T cells, endothelial cells, and can also be expressed in mononuclear–macrophage cell lines under pathogenic infection and cytokine induction.^13^ FGL2 has been reported to play an important role in some inflammatory diseases by enhancing proinflammatory activities of macrophages via mitogen-activated protein kinase (MAPK) signaling pathways.^14–16^ Furthermore, our previous study showed that FGL2 is expressed on microglia and increases simultaneously with proinflammatory cytokines in the fetal rat brains of intrauterine inflammation-induced perinatal WMI models.^17^ Similarly, we also found that intrauterine inflammation causes increased expression of FGL2 and proinflammatory cytokines in fetal brains of BALB/c background mice (Supplementary Fig. S1). Therefore, we hypothesized that FGL2 deficiency could protect against maternal inflammation-induced perinatal WMI by inhibiting the activation of MAPK pathway and proinflammatory polarization of microglia. In the present study, we aimed to explore the role and mechanisms of FGL2 in inflammation-induced perinatal WMI.

## Methods

### Animals

All animal care procedures were conducted according to the Chinese guidelines and regulations for experimental animals, and approved by the Institutional Animal Care and Use Committee, Tongji Medical College, Huazhong University of Science and Technology ([2015] IACUC Number: 2331). Wild-type (WT) BALB/c mice were purchased from the Experimental Animal Research Center of Hubei Province. The FGL2 knockout (KO) mice with BALB/c background were generated as described previously.^18,19^ The mice were housed in a specific pathogen-free facility at the temperature of 22 ± 2 °C, the humidity of 50–60%, under 12 h light/dark cycle, and were allowed standard mouse chow and sterile water ad libitum. Heterozygous mice were produced by crossing FGL2 KO mice and WT mice, and were further bred to obtain FGL2 KO mice and WT mice. The genotype was confirmed by PCR using primers as follows: FGL2, forward 5′-GATGAGGCTTCCTGGTT-3′ and reverse 5′-TTGCCACTTTGTTGTCC-3′.

Maternal lipopolysaccharide (LPS) administration-induced perinatal WMI model was conducted as previously described.^20^ Briefly, 100 µl sterile saline or 300 µg/kg LPS (*Escherichia coli*, serotype 055:B5; Sigma, USA) was intraperitoneally injected to WT or FGL2 KO dams on embryonic day (E) 16.5. The WT pups were obtained from the WT dams injected with saline or LPS. The FGL2 KO pups were obtained from the FGL2 KO dams. In order to obtain the fetal mice, the dams were anesthetized with pentobarbital 6 h after intraperitoneal injection, and the fetal mice were harvested by cesarean section. For the study of neonatal mice, pregnancies were allowed to proceed to term; then on postnatal day 1 (P1) and P7, neonatal mice were collected. After anesthesia and removal of skull and leptomeninges, brain weight was recorded, and neonatal brain tissues were obtained.

### Immunohistochemistry

After perfusion and fixation in 4% paraformaldehyde for 48 h and dehydration by gradient ethanol, the P7 neonatal brains were embedded in paraffin and cut into 4 µm sections. The sections were deparaffinized with xylene and rehydrated through graded ethanol. Endogenous peroxidase activity was eliminated by 3% hydrogen peroxide, and antigen retrieval was performed by boiled citrate buffer. The sections were incubated with rabbit polyclonal anti-MBP (1:50; Proteintech, Wuhan, China) overnight at 4 °C. Then, the sections were treated with streptavidin–biotin–horseradish peroxidase (SABC kit, Boster, Wuhan, China) according to the manufacturer’s instructions, and were counterstained with hematoxylin. Three discontinuous sections of each pup on the hippocampus level were selected. Three to five visual fields (×400 magnification) of each section in the periventricular region were obtained using an inverted microscope (Olympus, Tokyo, Japan) equipped with the Image-Pro Plus software (Version 6.0; Media Cybernetics, Silver Springs, MD, USA), and the percentage of positive staining area over the examined area were analyzed using the ImageJ software (Version 1.8.0; http://rsb.info.nih.gov/ij/; NIH, Bethesda, MD, USA). For each animal, the positive staining area (%) of sections were calculated and averaged as the final percentage.^21,22^

### Double immunofluorescent staining

Dehydration, antigen retrieval, and blocking were performed on the sections of P1 and P7 neonatal brains. Then, the sections were incubated immediately in mouse anti-ionized calcium-binding adaptor molecule-1 (Iba-1) monoclonal antibody (1:50; Servicebio, Wuhan, China) admixed with rabbit anti-CD86 polyclonal antibody (1:100; ABclonal, Wuhan, China), rabbit anti-CD206 polyclonal antibody (1:100; Proteintech, Wuhan, China), rabbit anti-inducible nitric oxide synthase (iNOS) polyclonal antibody (1:200; Servicebio, Wuhan, China), or rabbit anti-arginase 1 polyclobal antibody (1:200; ABclonal, Wuhan, China) overnight at 4 °C. On the second day, the sections were treated with Cy3-conjugated goat anti-rabbit IgG (H + L) and fluorescein isothiocyanate-conjugated goat anti-mouse IgG (H + L) (1:100; ABclonal, Wuhan, China) fluorescent secondary antibody at room temperature for 1 h, stained with DAPI (4′,6-diamidino-2-phenylindole) for 15 min. Images were collected through the above-mentioned inverted fluorescence microscope and processed using the Image-Pro Plus software. The number of immunopositive cells on the hippocampus level was counted by the ImageJ software. For each animal, three randomized sections were counted and averaged as the final cell number or percentage.

### Quantitative reverse transcription-PCR

Fresh E16.5 fetal brains, P1 neonatal brains, and P7 neonatal brains were immediately frozen with liquid nitrogen, and stored at −80 °C until use. Total RNA was extracted from brain tissues with TRIzol reagent (Invitrogen, Carlsbad, CA, USA) and quantified by UV spectrophotometer (Eppendorf, Hamburg, Germany). Then, cDNA was generated using PrimeScript RT Master Mix (Takara, Tokyo, Japan) according to the manufacturer’s protocol. Quantitative polymerase chain reaction was performed on CFX Connect Real-Time PCR Detection System (Bio-Rad, Hercules, CA, USA) using primers (Supplementary Table S1) and SYBR Premix EX Taq (Takara, Tokyo, Japan). The comparative threshold cycle (2^−∆∆CT^) method was used to analyze the expression values, normalizing to β-actin.

### Western blot

Protein from fetal and neonatal brains was extracted with ice-cold RIPA buffer (Boster, Wuhan, China) containing protease inhibitor and phosphorylated protease inhibitor. Equal amounts of protein were separated by 10% sodium dodecyl sulfate-polyacrylamide gel electrophoresis and then electrotransferred onto the 0.45 μm nitrocellulose membranes (Boster, Wuhan, China). The membranes were blocked with Odyssey blocking buffer (LI-COR Biosciences, Lincoln, NE, USA) for 1 h at room temperature and then incubated with primary antibody (Supplementary Table S2) overnight at 4 °C. The next day, the membranes were incubated with 1:15,000 goat anti-mouse IR Dye-800CW and goat anti-rabbit IR Dye-680RD fluorescent secondary antibody (LI-COR Biosciences, Lincoln, NE, USA) in the dark at room temperature for 1 h. Images were acquired using the Odyssey CLx Infrared Imaging System (LI-COR Biosciences, Lincoln, NE, USA) and analyzed by Image Studio software (version 5.0.21; LI-COR Biosciences, Lincoln, NE, USA).

### Statistical analysis

Statistical analysis was performed using SPSS (version 17.0; IBM, Armonk, NY, USA). All data were verified by the Shapiro–Wilk test and followed a normal distribution. One-way analysis of variance (ANOVA) with Tuke–Kramer post hoc test was used for multiple comparisons of normally distributed data. If homogeneity of variance was not satisfied, Welch’s ANOVA with Games–Howell post hoc test was used. Data are expressed as the means ± standard error of the mean (SEM) in the figures and are expressed as the means ± standard deviation (SD) in the table. The significant difference was defined as *P* < 0.05.

## Results

### KO of FGL2 protects against body weight loss induces by intrauterine inflammation

To obtain surviving pups, dams were intraperitoneally injected with 300 µg/kg LPS on E16.5. Offspring were divided into four groups: saline-treated WT mice, LPS-treated WT mice, saline-treated FGL2 KO mice, and LPS-treated FGL2 KO mice. We continuously monitored body weight and brain weight of offspring to investigate whether FGL2 deficiency can restore the effect of intrauterine inflammation on growth and brain development. There were no significant differences in body weight and brain weight among the four groups 6 h after the intervention (*n* = 12, Table 1). On P1, the body weight of LPS-treated WT mice was significantly lower than saline-treated WT mice (*P* = 0.029), while FGL2 KO mice were resistant to body weight changes caused by LPS (*P* = 0.019). LPS-treated WT mice showed only a slight increase in the brain-to-body weight ratio with no statistical difference compared with LPS-treated WT mice, since the brain weight of LPS-treated WT mice was lower than saline-treated WT mice (*n* = 9 for each group, Table 1). On P7, the body weight loss in WT mice (*P* = 0.016) caused by intrauterine inflammation was even more obvious with little reduction of brain weight, leading to a notable increase in brain-to-body weight ratio (*P* = 0.020). FGL2 KO can return weight loss (*P* = 0.041) and relative brain weight gain (*P* = 0.022) to normal (*n* = 9 for each group, Table 1).

**Table 1:** Body weight and brain weight of WT and FGL2 KO offspring after intrauterine inflammation.

Group	E16.5			P1			P7		
	Body weight (g)	Brain weight (g)	Brain/body weight (%)	Body weight (g)	Brain weight (g)	Brain/body weight (%)	Body weight (g)	Brain weight (g)	Brain/body weight (%)
WT/SAL	0.482 ± 0.054	0.044 ± 0.005	9.110 ± 0.780	1.463 ± 0.090	0.095 ± 0.008	6.487 ± 0.338	5.128 ± 0.240	0.263 ± 0.008	5.064 ± 0.190
WT/LPS	0.501 ± 0.064	0.045 ± 0.005	8.736 ± 0.969	1.362 ± 0.084*	0.092 ± 0.012	6.723 ± 0.637	4.584 ± 0.391*	0.254 ± 0.009	5.561 ± 0.295*
FGL2 KO/SAL	0.475 ± 0.084	0.044 ± 0.008	9.316 ± 0.764	1.437 ± 0.037	0.091 ± 0.002	6.359 ± 0.060	5.029 ± 0.128	0.252 ± 0.009	5.012 ± 0.167
FGL2 KO/LPS	0.487 ± 0.060	0.044 ± 0.007	9.242 ± 1.662	1.473 ± 0.054^†^	0.095 ± 0.003	6.429 ± 0.172	5.071 ± 0.390^†^	0.257 ± 0.007	5.087 ± 0.347^†^

Body weight and brain weight were measured and brain-to-body weight ratio was calculated on E16.5 (*n* = 12 for each group), P1 (*n* = 9 for each group), and P7 (*n* = 9 for each group). Data are expressed as mean ± SD.

*P < 0.05 WT/LPS versus WT/SAL.

^†^*P* < 0.05 FGL2 KO/LPS versus WT/LPS.

### KO of FGL2 improves hypomyelination induced by maternal inflammation

**Fig. 1:**
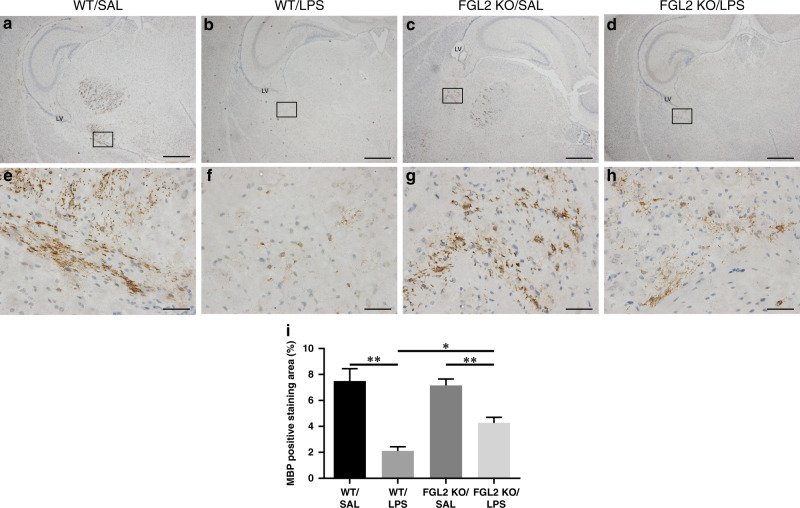
Expression of MBP in WT and FGL2 KO mice brains after intrauterine inflammation. MBP immunohistochemical staining was used to detect the myelination of white matter among the four groups on P7 (**a**–**d** low magnification, scale bars: 500 µm; **e**–**h**: high magnification, scale bars: 50 µm) (*n* = 6 for each group). On the hippocampus level, MBP positive staining was mainly observed in the periventricular region. The percentage of MBP positive staining area (**e**) are presented as mean ± SEM; *P < 0.05 and **P < 0.01.

Hypomyelination is one of the main features of neonatal WMI. We further explored the impact of intrauterine inflammation on myelination. Major basic protein (MBP) is a marker of oligodendrocytes and plays an important role in myelination. To investigate whether FGL2 KO has a protective effect on hypomyelination, we examined the expression of MBP in the brain tissues of P7 neonatal mice by immunohistochemistry (*n* = 6 for each group) and found that the most prominent loss of MBP occurred in the periventricular region of LPS-treated WT mice (Fig. 1b, f). The percentage of MBP positive staining area significantly reduced in LPS-treated WT mice compared with saline-treated WT mice (*P* = 0.007, Fig. 1i). LPS-treated FGL2 KO mice also showed a significant reduction in the expression of MBP compared with saline-treated FGL2 KO mice (*P* = 0.006, Fig. 1d, h). However, compared with LPS-treated FGL2 WT mice, the expression of MBP in LPS-treated FGL2 KO mice increased with statistical difference (*P* = 0.011).

### KO of FGL2 inhibits the expression of cerebral proinflammatory cytokines

**Fig. 2:**
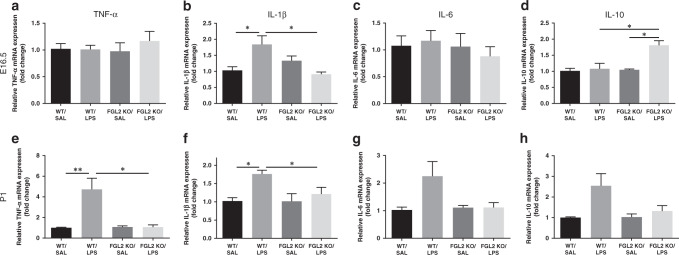
Expression of cytokines in WT and FGL2 KO mice brains after intrauterine inflammation. The mRNA levels of the proinflammatory cytokines TNF-α, IL-1β, IL-6, and anti-inflammatory cytokines IL-10 on E16.5 (**a**–**d**) and P1 (**e**–**g**) (*n* = 6 for each group) were detected by qPCR. Data are expressed as mean ± SEM; *P < 0.05 and **P < 0.01.

Cytokine is one of the key regulators of inflammatory response, closely linking intrauterine inflammation with neonatal WMI. To assess the impact of FGL2 on cytokines, we compared the mRNA levels of the proinflammatory cytokines interleukin-1β (IL-1β), tumor necrosis factor-α (TNF-α), and IL-6, as well as the anti-inflammatory cytokines IL-10, in WT and FGL2 KO mice (*n* = 6 for each group). In fetal mice, LPS treatment significantly induced the expression of IL-1β in WT mice (*P* = 0.015), while FGL2 deficiency inhibited the induction of IL-1β (*P* = 0.011, Fig. 2b). Meanwhile, the level of anti-inflammatory cytokine IL-10 was significantly increased in LPS-treated FGL2 KO fetal mice compared with saline-treated FGL2 KO fetal mice (*P* = 0.011) and LPS-treated WT fetal mice (*P* = 0.030, Fig. 2d). No significant changes of TNF-α and IL-6 levels (Fig. 2a, c) were observed between saline- and LPS-treated WT fetal mice. For neonatal mice, we observed a significantly increased expression of TNF-α (*P* = 0.006, Fig. 2e) and IL-1β (*P* = 0.014, Fig. 2f) in LPS-treated WT mice compared with saline-treated WT mice. KO of FGL2 statistically reduced the expression of proinflammatory cytokines TNF-α (*P* = 0.017) and IL-1β (*P* = 0.026). IL-6 and IL-10 levels were slightly increased in LPS-treated WT neonatal mice, but not significantly (Fig. 2g, h). On P7, there was no significant difference in the expression of TNF-α, IL-1β, IL-6, and IL-10 among the four groups (Supplementary Fig. S2).

### KO of FGL2 decreases M1 microglial polarization and increases M2 microglial polarization

**Fig. 3:**
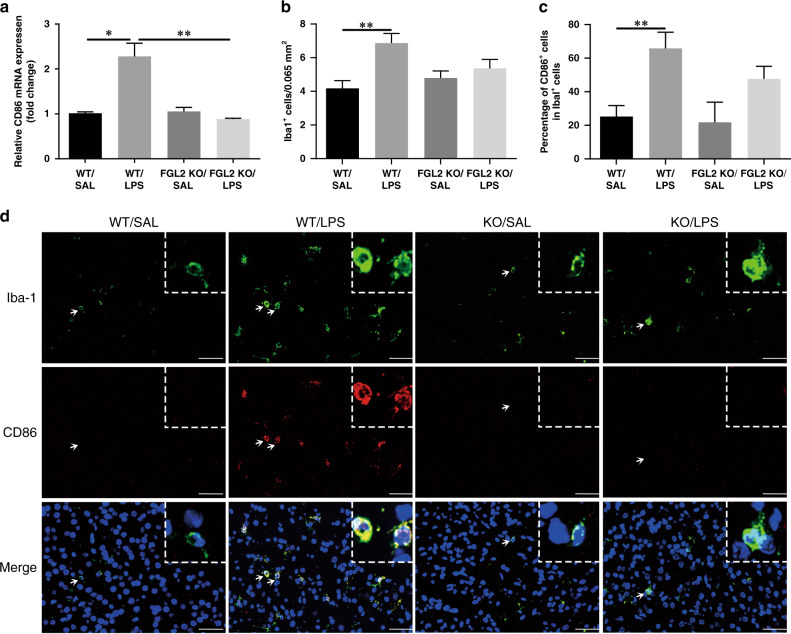
Effects of intrauterine inflammation on M1 activation of microglia in WT and FGL2 KO mice on P1. CD86 mRNA expression levels were detected by qPCR (**a**) (*n* = 6 for each group). Immunofluorescence double staining of Iba-1 (green) and CD86 (red) was shown as proinflammatory (M1) microglia (**d**). Arrow indicates the typical cells. Scale bars: 50 μm. Immunopositive cells were semi-quantified, and were expressed as percentages (**b**, **c**) (*n* = 6 for each group). Data are presented as mean ± SEM; *P < 0.05 and **P < 0.01.

**Fig. 4:**
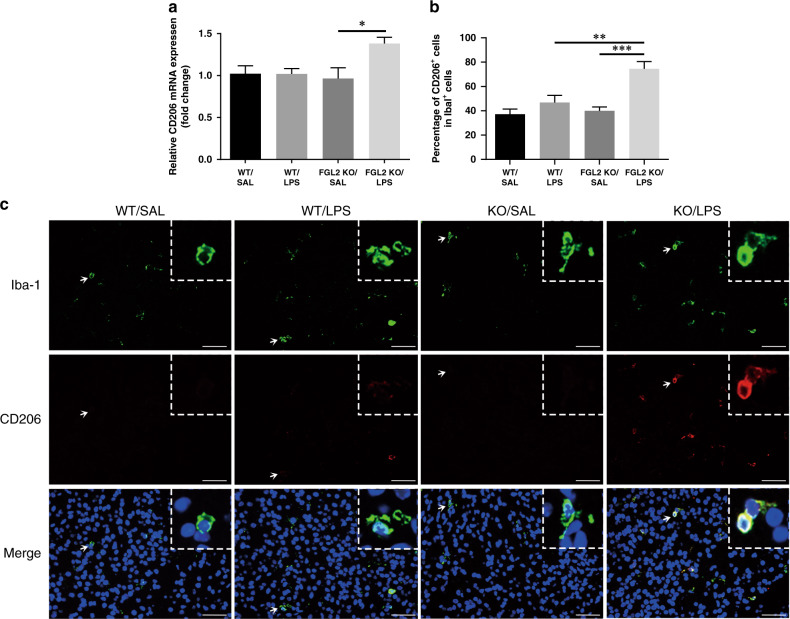
Effects of intrauterine inflammation on M2 activation of microglia in WT and FGL2 KO mice on P1. CD206 mRNA expression level were detected by qPCR (**a**) (*n* = 6 for each group). Fluorescence double staining of Iba-1 (green) and CD206 (red) was shown as anti-inflammatory (M2) microglia (**c**). Arrow indicates the typical cells. Scale bars: 50 μm. The percentage of CD206+ cells in Iba-1+ cells was analyzed (**b**) (*n* = 6 for each group). Data are presented as mean ± SEM; *P < 0.05, **P < 0.01 and ***P < 0.001.

Microglia are immune cells, which can be activated by cytokines and differentiated into proinflammatory (M1) or anti-inflammatory (M2) type. To understand the effect of FGL2 KO on microglial polarization, we examined the microglial phenotypes (*n* = 6 for each group). The expression of M1 marker CD86 (Supplementary Fig. S3A) and M2 marker CD206 (Supplementary Fig. S3D) showed no significant difference among the four fetal groups. On P1, the mRNA level of CD86 was significantly increased in LPS-treated WT neonatal brains (*P* = 0.014) compared with saline-treated control mice, meanwhile FGL2 KO blunted the induction of CD86 (*P* = 0.008, Fig. 3a). Iba-1 is one of the microglial marker. Colocalization of Iba-1 and CD86 by double immunofluorescence staining, further confirmed that LPS-treated WT mice had higher expression of CD86 on microglia than saline-treated WT mice (*P* = 0.009, Fig. 3c, d). However, LPS intervention did not significantly increase CD86 expression in FGL2 KO mice. Similarly, LPS intervention increased the mRNA level (*P* = 0.031) and protein level (*P* < 0.001) of another M1 marker iNOS in WT mice (Supplementary Fig. S3B, C, G), but had no significant effect on KO mice. Moreover, FGL2 KO could increase the mRNA level of M2 marker CD206 in LPS-treated FGL2 KO neonatal mice compared with saline-treated FGL2 KO mice (*P* = 0.034, Fig. 4a). Immunofluorescence double staining of Iba-1 and CD206 also found that FGL2 deficiency could increase the number of CD206 and Iba-1 double-positive microglia (*P* = 0.005 versus WT/LPS, *P* < 0.001 versus FGL2 KO/SAL, Fig. 4b, c). These results suggested that FGL2 deficiency can reduce proinflammatory polarization and increase anti-inflammatory polarization of microglia.

On P7, there was no significant difference in the expression of CD86 and iNOS (Supplementary Fig. S4A, B, F, G). Compared with saline-treated WT mice, the mRNA (*P* = 0.043) and protein (*P* = 0.007) levels of M2 marker CD206 significantly increased in LPS-treated WT mice (Supplementary Fig. S4C, H). In FGL2 KO mice, the percentage of CD206+ cells in Iba-1+ cells (*P* = 0.013, Supplementary Fig. S4H), but not the mRNA level was significantly increased under LPS intervention. Therefore, we hypothesized that FGL2 deficiency may have a prolonging effect on microglial polarization.

### KO of FGL2 inhibits p38 MAPK and JNK pathway activation

**Fig. 5:**
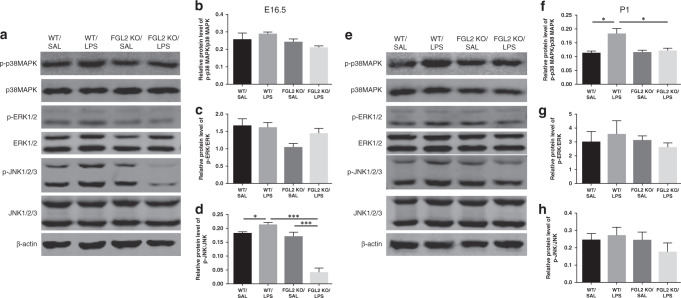
Expression of MAPK pathway key protein in WT and FGL2 KO mice brains after intrauterine inflammation. On E16.5 (**a**–**d**) and P1 (**e**–**h**), the total and phosphorylated protein levels of p38 MAPK, ERK, and JNK were evaluated by Western blotting (*n* = 6 for each group). The relative phosphorylated protein levels were calculated as the ratio of phosphorylated protein to total protein and expressed as the mean ± SEM; *P < 0.05 and ***P < 0.001.

MAPK pathway plays an important regulatory role in the inflammatory cascade and microglia polarization. FGL2 has been reported to be associated with the MAPK pathway in inflammatory diseases. Thus, we measured protein levels of p38 MAPK, extracellular signal-regulated kinase (ERK), and c-Jun N-terminal kinase (JNK) to determine whether FGL2 KO altered the activation of MAPK pathway in the intrauterine inflammation-induced neuroinflammation (*n* = 6 for each group). In fetal brains, the phosphorylated JNK protein level in the LPS-treated WT mice was statistically increased compared with saline-treated WT mice (*P* = 0.036, Fig. 5a, d). FGL2 KO attenuated the phosphorylation of JNK protein induced by intrauterine inflammation (*P* < 0.001). The p38 MAPK and ERK protein did not change significantly after LPS intervention (Fig. 5b, c). In neonatal brains, compared with saline-treated WT mice, the expression of phosphorylated p38MAPK protein increased significantly in LPS-treated WT mice (*P* = 0.012), while FGL2 KO suppressed the activation of p38 MAPK protein (*P* = 0.030, Fig. 5e, f). The change in trend of JNK phosphorylation among the four groups of neonatal mice was similar to those of fetal mice, but did not reach statistical significance (Fig. 5h). The level of ERK protein still had no obvious difference among the four groups (Fig. 5g).

## Discussion

Intrauterine infection and inflammation is a risk factor for perinatal WMI.^23^ Although much progress has been made in related research, the cellular and molecular mechanisms remain unclear and clinically available therapies are lacking. Our previous study showed that the expression of FGL2 and proinflammatory cytokines simultaneously elevate in the fetal brains after maternal inflammation, and FGL2 expression is localized on microglia.^17^ In this study, we confirmed that FGL2 deficiency can shift microglial polarization to anti-inflammatory type through the MAPK pathway, decrease the proinflammatory cytokines, reduce the impairment of the oligodendrocyte maturation, and eventually improve WMI induced by intrauterine inflammation.

FGL2 has been identified as a member of the fibrinogen-related protein superfamily, and is capable of procoagulant activity. Inflammatory cytokines can induce FGL2 expression.^24,25^ In turn, FGL2 can also promote the release of cytokines through prothrombin activation and fibrin deposition.^26,27^ Inflammation and coagulation coexist and amplify each other, leading to the development of multiple diseases. Previous studies found that FGL2 expression is closely related to the brain inflammatory injury in cerebral hemorrhage diseases.^11,12^ Leviton and Dammann^9^ suggested that activated coagulation factors contribute to neonatal white matter damage by exacerbating inflammation. Our study revealed that FGL2 deficiency can reduce the expression of proinflammatory cytokines IL-1β, TNF-α, and IL-6, limiting the inflammatory cascade. Sylvie and colleagues^28^ used a specific IL-1 receptor antagonist to protect most behavioral and histological parameters, providing evidence that IL-1 is the initiator of the molecular cascades leading to brain damage. Similarly, our study suggested that IL-1β was inhibited earlier than TNF-α and IL-6. Meanwhile, FGL2 deficiency rapidly promoted the expression of the anti-inflammatory cytokine IL-10, which had a neuroprotective effect. The expression of IL-10 in LPS-treated WT neonatal mice was slightly increased, possibly due to the negative feedback regulation. There was a clinical investigation, which showed that the relative balance between pro- and anti-inflammatory cytokines is a predictor of the offspring birth weight.^29^ Our study demonstrated that neonatal WT mice exposed to maternal inflammation had a lower body weight than those that were not exposed. This may be partly due to the higher levels of proinflammatory cytokines. FGL2 KO regulates the balance of pro- and anti-inflammatory cytokines, protecting against neonatal body weight loss.

In inflammatory diseases outside nerve system, FGL2 expression is increased on macrophages. Upregulation of FGL2 mediates macrophage activation, while silencing of FGL2 blocks macrophage activation and the release of proinflammatory cytokines.^15^ Furthermore, Li et al.^30^ demonstrated that FGL2 deficiency shifts the macrophage phenotype from proinflammatory (M1) to anti-inflammatory (M2) pattern. We found that FGL2 deficiency can also alter the phenotype of microglia, the brain-resident macrophages. Intrauterine inflammatory exposure results in M1 microglia polarization. Proinflammatory microglia may release proinflammatory cytokines and aggravate brain damage. The KO of FGL2 reversed the microglial phenotype and polarized microglia to M2 type. The effects may last for a while. In general, FGL2 deficiency can inhibit proinflammatory polarization of microglia and promote anti-inflammatory polarization.

LPS-induced microglial activation is dependent on the MAPK pathway. Some compounds exert an inhibitory effect on MAPK activation, suppressing the M1 polarization of microglia and shifting microglial polarization to M2 type.^31–33^ Moreover, increased FGL2 expression is associated with higher protein phosphorylation in the MAPK pathway.^13^ In order to explore the mechanism of FGL2 KO on microglial polarization, we investigated the activation of the MAPK pathway. We revealed that FGL2 deficiency significantly reduced the phosphorylation of p38 MAPK protein in neonatal mice. For fetal mice, the phosphorylation of JNK protein induced by intrauterine inflammation was strongly inhibited by FGL deficiency, which was consistent with the increased expression of the anti-inflammatory cytokines IL-10. We speculated that FGL2 KO not only inhibits the inflammatory cascade but also further increases the generation of anti-inflammatory factors through the JNK pathway, playing a neuroprotective role. However, FGL2 KO regulates microglial polarization and the expression of cytokines mainly through the p38 MAPK and JNK pathways, but not ERK. This is probably because ERK pathway is not only affected by FGL2 but also other signaling pathways. This result has been supported by a previous report. Both JNK and p38 MAPK pathways are important in the induction of TNF-α in LPS-stimulated microglia, while ERK appears to be less impactful in the induction.^34^

WMI is characterized by oligodendrocyte damage and hypomyelination. Coculture of microglia with oligodendrocyte progenitor cells showed that substances such as cytokines produced by LPS-activated microglia would affect the survival and maturity of oligodrocytes.^35^ Our study showed that LPS intervention reduced the expression of MBP in FGL2 KO mice compared with saline-treated FGL2 KO mice, but not as severely as WT mice. The MBP expression level of LPS-treated FGL2 KO mice was significantly higher than that of LPS-treated WT mice. This indicates that although there are other factors that lead to brain damage, FGL2 KO can still promote the maturation of oligodendrocytes and reduce the apoptosis of oligodendrocytes. FGL2 deficiency can improve WMI by alleviating inflammation-induced hypomyelination.

The limitation of this study is the use of systemic FGL2 KO mice. It is not clear whether the improvement in WMI is due to reduced systemic inflammation, local neuroinflammation, or both. Conditional KO mice or in vitro experiments are needed for further research. Second, WT and FGL2 KO offspring were bred by WT and FGL2 KO dams, respectively, rather than from the same heterozygous dams. It can be seen that there were differences between WT/LPS and WT/SAL, while KO/LPS and KO/SAL were not significantly different, which indirectly indicates the improvement effect. The comparison of KO/LPS and WT/LPS cannot explain the role of FGL2 in perinatal mice, but it could, to a certain extent, show the overall effect of FGL2 deficiency on intrauterine inflammation in dams and inflammatory response in pups together. Similar to the Foerster et al.^36^ study, we found that the heterozygous mating produced few homozygotes, so dams with different genotypes were selected. In the future, we could further explore the mechanism of FGL2 through embryo transfer technology^37^ and coculture of FGL2 KO microglia and oligodendrocytes in vitro. Furthermore, the long-term brain function of offspring and the role of FGL2 in endothelial cells and procoagulant cascade require further investigated.

In conclusion, by this study, we found that FGL2 deficiency promotes anti-inflammatory polarization of microglia, reduces inflammatory cascade, and alleviates WMI via the inhibition of p38 MAPK and JNK pathways. FGL2 could be a novel molecular target for the prevention of intrauterine inflammation-induced WMI.

## Supplementary information

Supplementary Material
