# Supplementary material for: Intrauterine inflammation induced white matter injury protection by fibrinogen-like protein 2 deficiency in perinatal mice
Source: Pediatr Res. 2020 Oct 19;89(7):1706–14. doi: 10.1038/s41390-020-01211-w (PMC8249236; doi:10.1038/s41390-020-01211-w)
Supplement: Supplementary file 1 — Supplementary Material [file 41390_2020_1211_MOESM1_ESM.docx]

**Intrauterine inflammation induced white matter injury protection by fibrinogen-like protein 2 deficiency in perinatal mice**

**SUPPLEMENTARY MATERIAL**


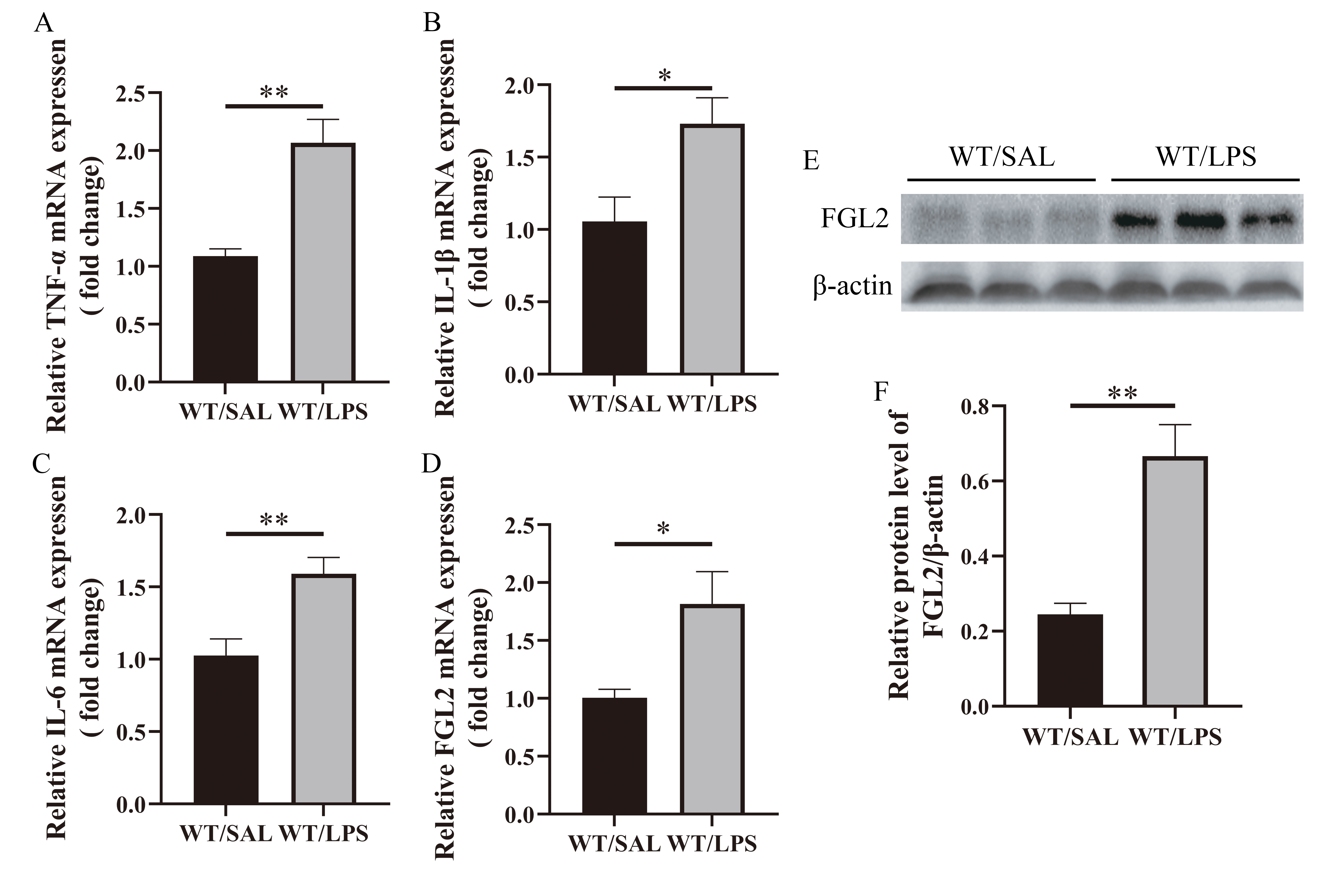


**Supplementary Figure S1. Expression of proinflammatory cytokines and FGL2 in the fetal mouse brains.** WT pregnant mice were intraperitoneally injected with saline or 300 μg/kg LPS on E16.5. On E18.5, the mRNA levels of proinflammatory cytokines TNF-α (A), IL-1β (B), and IL-6 (C) in fetal brains were measured by qPCR. FGL2 mRNA (D) and protein (E-F) expression levels were analyzed by qPCR and Western Blot, respectively. Data are expressed as mean ± SEM; **P* < 0.05, ***P* < 0.01.

**Supplementary Table S1**

**Primer sequences for RT-qPCR**

| Symbol | Forward primer sequence | Reverse primer sequence |
| --- | --- | --- |
| β-actin | 5’-GTGACGTTGACATCCGTAAAGA-3’ | 5’-GTAACAGTCCGCCTAGAAGCAC-3’ |
| TNF-α | 5’-CAGGCGGTGCCTATGTCTC-3’ | 5’-CGATCACCCCGAAGTTCAGTAG-3’ |
| IL-1β | 5’-ATGCCACCTTTTGACAGTGATG-3’ | 5’-TGTGCTGCTGCGAGATTTGA-3’ |
| IL-6 | 5’-TAGTCCTTCCTACCCCAATTTCC-3’ | 5’-TTGGTCCTTAGCCACTCCTTC-3’ |
| IL-10 | 5’-GCTCTTACTGACTGGCATGAG-3’ | 5’-CGCAGCTCTAGGAGCATGTG-3’ |
| CD86 | 5’-TTGTGTGTGTTCTGGAAACGGAG-3’ | 5’-AACTTAGAGGCTGTGTTGCTGGG-3’ |
| CD206 | 5’-CTCTGTTCAGCTATTGGACGC-3’ | 5’-CGGAATTTCTGGGATTCAGCTTC-3’ |
| iNOS | 5’-ACATCGACCCGTCCACAGTAT-3’ | 5’-CAGAGGGGTAGGCTTGTCTC-3’ |
| Arg1 | 5’-GTACATTGGCTTGCGAGACG-3’ | 5’-GGCCTTTTCTTCCTTCCCAG-3’ |

**Supplementary Table S1.** **RT-qPCR primers.** The primers we used in this study. The primers were designed by BLAST and were synthesized by Tsingke (Beijing, China).

**Supplementary Table S2**

**Primary antibody for Western Blot**

| Antibody name | Supplier | Catalog number | Dilution |
| --- | --- | --- | --- |
| beta Actin Mouse Monoclonal antibody | Proteintech, Wuhan, China | 60008-1-Ig | 1:2000 |
| p38 MAPK (D13E1) XP® Rabbit mAb | CST, Danvers, MA, USA | 8690 | 1:1000 |
| Phospho-p38 MAPK (Thr180/Tyr182) (28B10) Mouse mAb | CST, Danvers, MA, USA | 9216 | 1:1000 |
| p44/42 MAPK (Erk1/2) (137F5) Rabbit mAb | CST, Danvers, MA, USA | 4695 | 1:1000 |
| Phospho-p44/42 MAPK (Erk1/2) (Thr202/Tyr204) (D13.14.4E) XP® Rabbit mAb | CST, Danvers, MA, USA | 4370 | 1:1000 |
| JNK Mouse Monoclonal antibody | Proteintech, Wuhan, China | 66210-1-Ig | 1:10000 |
| Recombinant Anti-JNK1 + JNK2 + JNK3 (phospho T183+T183+T221) antibody | Abcam, Cambridge, MA, USA | ab124956 | 1:1000 |

**Supplementary Table S2.** The primary antibodies we used for western blot in this study.


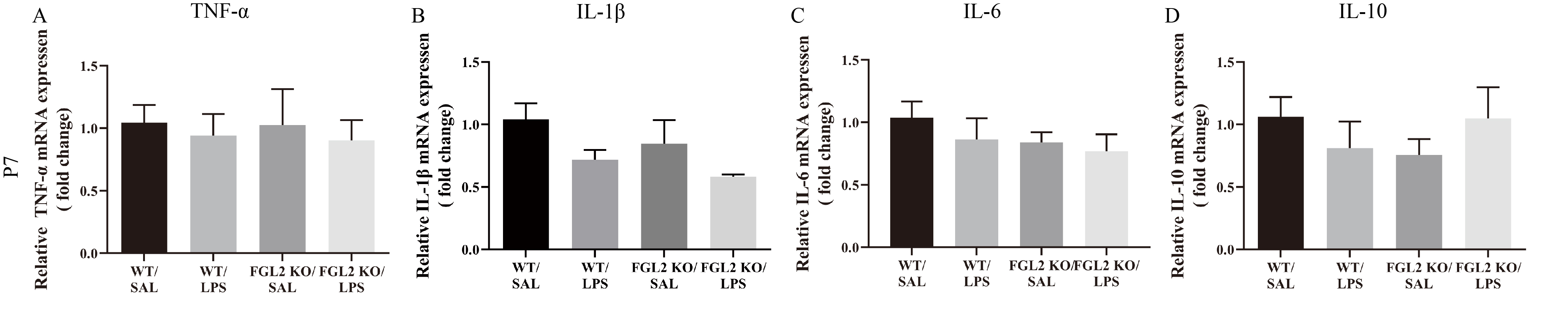


**Supplementary Figure S2. Expression of cytokines in WT and FGL2 KO mice brains after intrauterine inflammation on P7.** The mRNA levels of the proinflammatory cytokines TNF-α (A), IL-1β (B), IL-6 (C) and anti-inflammatory cytokines IL-10 (D) on P7 (*n* = 6 for each group) were detected by qPCR. Data are expressed as mean ± SEM.

**Supplementary Figure S3. Effects of intrauterine inflammation on M1 and M2 activation of microglia in WT and FGL2 KO mice.** The mRNA expression levels of CD86 (A), CD206 (D) in fetal mice and iNOS (B), Arg1 (E) in P1 mice were detected by qPCR (*n* = 6 for each group). On P1, immunofluorescence double staining of Iba1 (green) and iNOS (red) was shown as proinflammatory (M1) microglia (G). Arrow indicates the typical cells. Scale bars: 50 um. Immunopositive cells were semi-quantified, and expressed as percentage (C). The percentage of Arg1^+^ cells in Iba1^+^ cells was also analyzed (F) (*n* = 6 for each group). Negative control (H). Data are presented as mean ± SEM; **P* < 0.05, ****P* < 0.001.


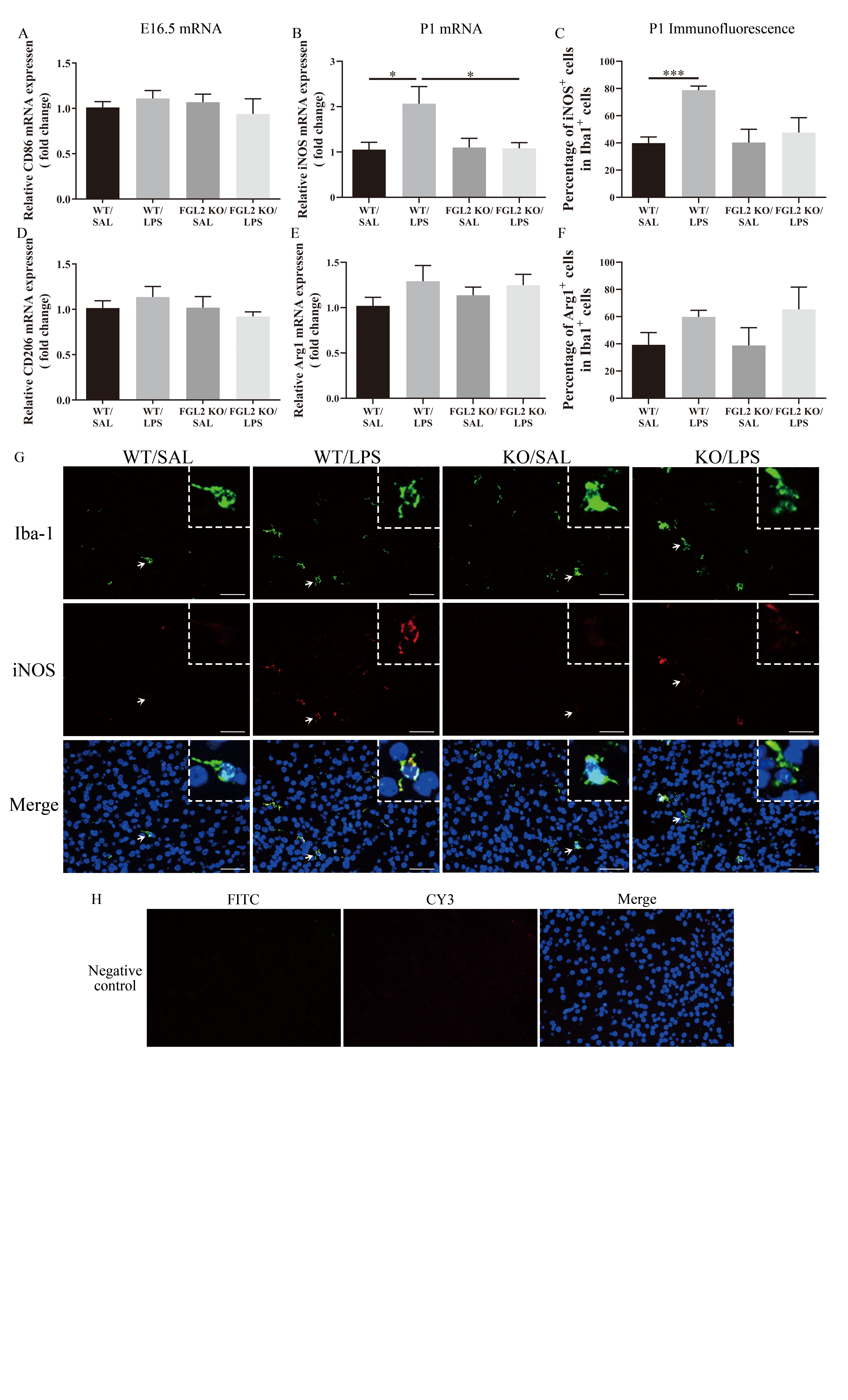

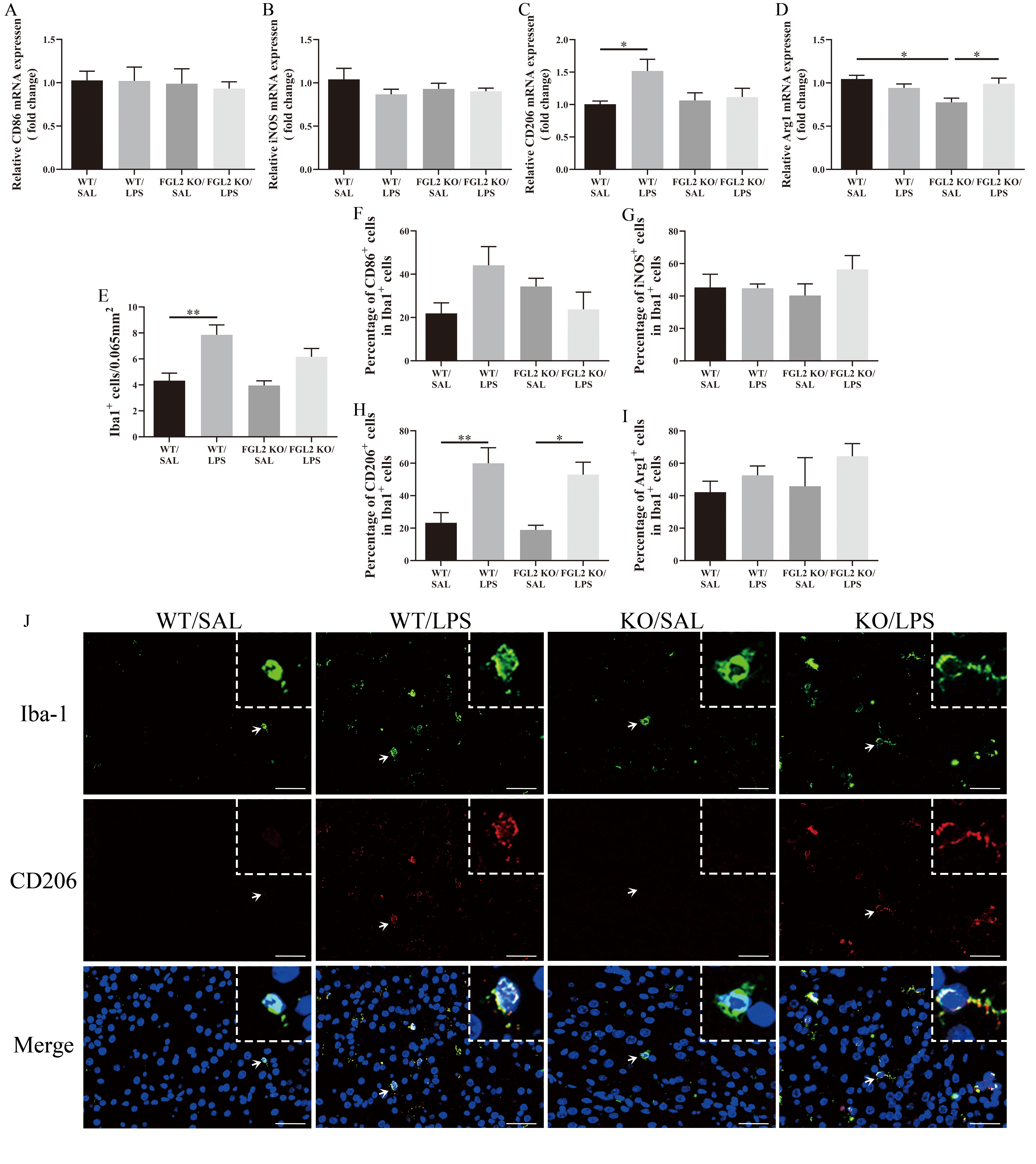


**Supplementary Figure S4. Effects of intrauterine inflammation on M1 and M2 activation of microglia in WT and FGL2 KO mice on P7.** The mRNA expression levels of CD86 (A), iNOS (B), CD206 (C) and Arg1 (D) were detected by qPCR (*n* = 6 for each group). Immunofluorescence double staining of Iba1 (green) and CD206 (red) was shown as proinflammatory (M2) microglia (J). Arrow indicates the typical cells. Scale bars: 50 um. Immunopositive cells were semi-quantified, and expressed as percentage (H). The percentage of CD86^+^ cells (F), iNOS^+^ cells (G), and Arg1^+^ cells (I) in Iba1^+^ cells were also analyzed (n=6 for each group). Data are presented as mean ± SEM; **P* < 0.05, ***P* < 0.01.
